# Tyrosine catabolites influence SKN-1 signaling in a model of Type I Tyrosinemia

**DOI:** 10.17912/micropub.biology.000577

**Published:** 2022-06-02

**Authors:** Talha F Siddiqi, Phillip A Frankino, Andrew Dillin

**Affiliations:** 1 Howard Hughes Medical Institute University of California, Berkeley, CA 94720; 2 California Institute for Regenerative Medicine, Berkeley, CA 94720 USA; 3 Department of Molecular and Cell Biology, University of California, Berkeley

## Abstract

Hereditary Tyrosinemia Type 1 (HT1) is a rare genetic disease that results from mutations of the tyrosine catabolism enzyme fumarylacetoacetate hydrolase (FAH) for which there is currently no cure. HT1 is successfully modeled in the nematode *C. elegans* , via mutations in the fumarylacetoacetate hydrolase ( *fah-1* ) resulting in abnormalities in body size, intestinal degradation, and activation of SKN-1/NRF2. Previous work has shown that body size and intestinal phenotypes in this model may occur through the buildup of toxic tyrosine catabolites, although the mechanism by which SKN-1 becomes activated remains elusive. Here, we confirm previous findings that phenotypes in the HT1 model are dependent on upstream enzymes in this pathway. Notably, we find that *fah-1* mediated SKN-1 activation is dependent on the upstream enzymes in this pathway, suggesting that an accumulation of tyrosine catabolites influence SKN-1 activity. Finally, we report that SKN-1 responds to knockdown of multiple tyrosine catabolism enzymes, suggesting that multiple catabolites act as signaling inputs to SKN-1 and that *C. elegans* are an appropriate model to study diseases related to tyrosine catabolism.

**Figure 1. Intermediates of the Tyrosine catabolism pathway affect SKN-1 signaling:**
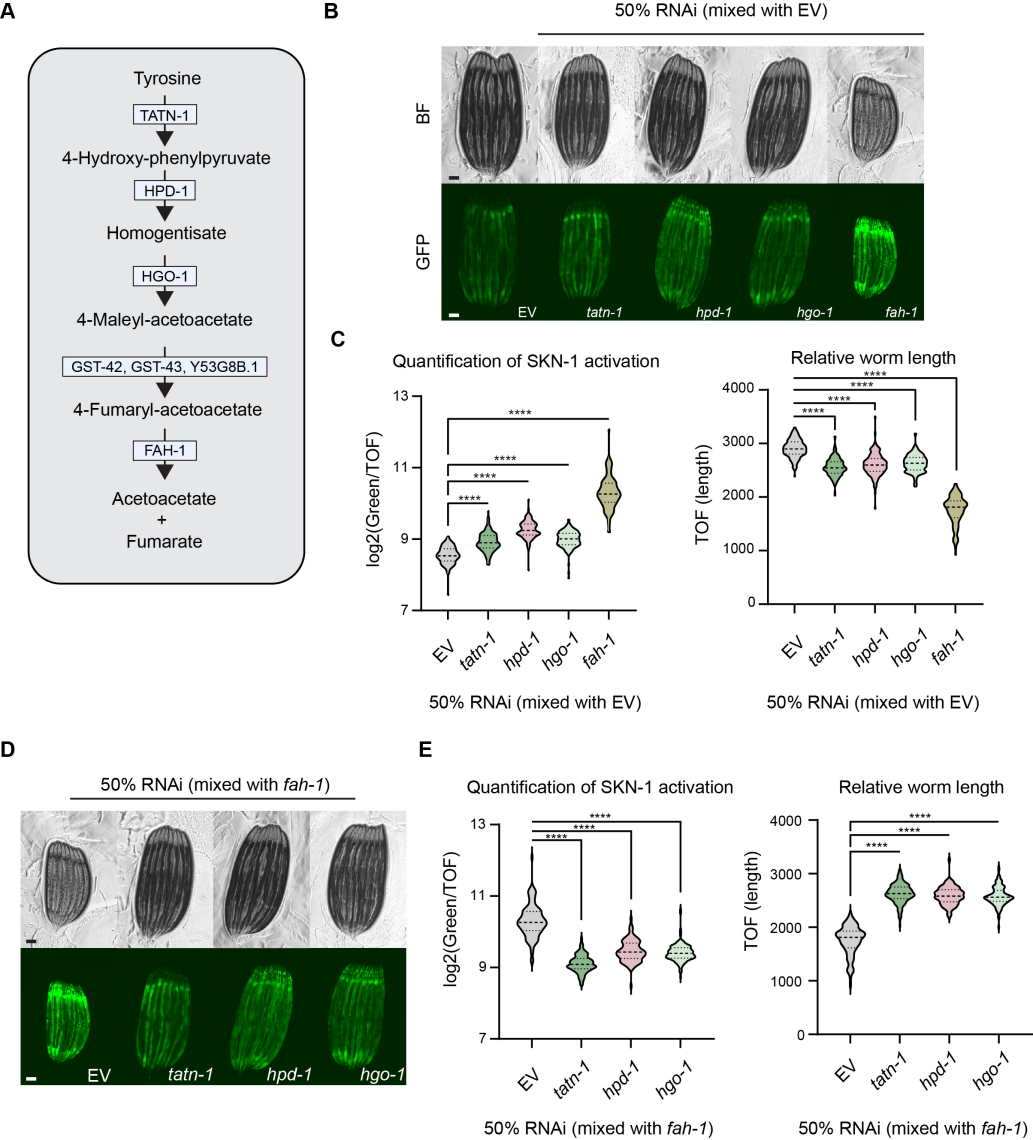
( **A** ) Schematic of the tyrosine catabolism pathway in *C. elegans* based on the latest version of the KEGG database **(B)** Fluorescent images of SKN-1 reporter animals ( *gst-4::GFP)* grown on bacteria expressing control (EV) or indicated RNAi mixed 1:1 with EV, scale bar 100 μm. **(C)** Quantification of SKN-1 activation and relative worm length using a COPAS biosorter. Data is representative of n=3 trials, n>100 animals; **** = P < 0.0001 using a one-way ANOVA **(D)** Fluorescent images of SKN-1 reporter animals ( *gst-4::GFP* ) grown on bacteria expressing indicated RNAi mixed 1:1 with *fah-1* RNAi, scale bar 100 μm. **(E)** Quantification of SKN-1 activation and relative worm length using a COPAS biosorter. Data is representative of n=3 trials, n>96 animals; **** = P < 0.0001 using a one-way ANOVA.

## Description

Type 1 Tyrosinemia (HT1) is a rare genetic disorder stemming from mutations in the tyrosine catabolism enzyme fumarylacetoacetate hydrolase (FAH), for which there exists no available cure (Russo et al., 2001) . The pathophysiological manifestations of HT1 include failure to thrive, liver and renal failure in addition to a predisposition for developing hepatocellular carcinoma (Jones et al., 2020) . HT1 has been successfully modeled in *C. elegans* , where animals mutant for *fah-1,* the nematode ortholog of FAH, show severe phenotypes of decreased body size, intestinal damage, reduced fertility, premature death, and activation of ER and oxidative stress pathways (Fisher et al. 2008). Manifestations of some of these phenotypes, such as intestinal damage and decreased body size, were found to depend on the enzymes upstream of *fah-1* . These findings are in line with the discovery that the buildup of multiple metabolites, mainly maleylacetoacetate (MAA) and fumarylacetoacetate (FAA), result in the formation of succinylacetoacetate (SAA) and succinylacetone (SA), driving disease pathology in humans (Lindblad et al., 1977; Morrow and Tanguay, 2017) .

Tyrosine catabolism follows a five-step pathway involving multiple enzymes and intermediates, leading to the formation of acetoacetoacetate and fumarate; these molecules are important for fatty acid synthesis and the tricarboxylic acid cycle (Figure 1A) (Ogata et al., 1999) . Previous work from our lab has demonstrated that SKN-1 can respond to perturbations of multiple amino acid catabolism pathways (Frankino et al., 2022) and verified that knockdown of *fah-1* induces a SKN-1 dependent reporter strain ( *gst-4p::GFP* ), a general readout of the oxidative stress response (Link and Johnson, 2002) . Additionally, work from others has shown that this activation of *gst-4p::GFP* upon *fah-1* knockdown is completely dependent on SKN-1 (Ferguson et al., 2010). To better understand the effects of disrupting tyrosine catabolism on SKN-1 activation, we used RNAi to knockdown multiple enzymes in this pathway and assessed SKN-1 activity using these reporter animals. We find that knockdown of *tatn-1, hgo-1, hpd-1,* and *fah-1* significantly activates SKN-1 in reporter animals (Figure 1B,C). Additionally, when these enzymes are knocked down, we find a significant decrease in animal body size with *fah-1* knockdown having the most severe effect. Since *fah-1* knockdown generated the most severe effects on body size, and earlier experiments showed a dependence on upstream enzymes for this phenotype, we sought to understand if SKN-1 activation was also dependent on upstream enzymes. We observed that tandem knockdown of *fah-1* and either *tatn-1, hgo-1 or hpd-1* significantly rescued body size and suppressed SKN-1 activation in comparison to *fah-1* knockdown alone (Figure 1E,D). These data both confirm that the buildup of tyrosine catabolites affect physiological phenotypes of this model and find that they may induce an oxidative stress response driven by SKN-1.

Here we report that, in a genetic model of HT1, where the accumulation of tyrosine catabolites is thought to drive pathology, SKN-1 activation is dependent on upstream tyrosine catabolism enzymes, suggesting that tyrosine catabolites also drive this response. These findings are in line with the understanding that, in HT1, MAA and FAA form SAA and SA which can cause oxidative stress and damage to the cell to drive disease pathology (Morrow and Tanguay, 2017) . Indeed, it has been previously demonstrated that expression of *aip-1* , another SKN-1 target, is induced by addition of terminal tyrosine metabolite SA (Ferguson et al., 2010). Here, we also found that knockdown of other tyrosine catabolism enzymes *tatn-1* , *hgo-1,* and *hpd-1* activate a SKN-1 reporter strain which may be indicative of an oxidative stress response. Indeed, *tatn-1* has been shown to be controlled by SKN-1 and metabolize *m* -tyrosine, a molecule that is thought to contribute to oxidative stress in both plants and animals (Ipson et al., 2019) . Thus, knockdown of *tatn-1* may induce oxidative stress via buildup of *m-* tyrosine. Furthermore, *hgo-1* and *hpd-1* knockdown may activate SKN-1 via accumulation of their upstream catabolites and thus represent new models to study their associated diseases (alkaptonuria and tyrosinemia type III, respectively).

## Methods

***C. elegans* maintenance and RNAi knockdown**

All *C. elegans* strains were maintained at 15°C on NGM plates with OP50 *E. coli* B strain. All experiments were performed at 20°C on RNAi plates (NGM agar, 1 mM IPTG, 100 μg/mL carbenicillin) with HT115 *E. coli* K12 strain bacteria containing the RNAi plasmid pL4440 empty vector as a negative control (EV) or containing sequence to synthesize a double-stranded RNA against a target gene. Mixed RNAi experiments were performed by growing the indicated RNAi expressing bacteria to saturation and combining 1:1 before plating. All RNAi constructs were isolated from the Vidal or Ahringer RNAi library and sequence verified with the M13 forward primer before using, sequence below.

M13F: 5’ - GTAAAACGACGGCCAGT - 3’

For all experiments, eggs were obtained using a standard bleaching protocol (1.8% sodium hypochlorite and 0.375 M KOH) and arrested at the L1 stage overnight in M9 (22 mM KH _2_ PO _4_ monobasic, 42.3 mM Na _2_ HPO _4_ , 85.6mM NaCl, 1 mM MgSO _4_ ) without food for synchronization. The next day, synchronized L1 animals were placed on HT115 bacteria and grown until day 1 of adulthood.

**Fluorescence imaging and quantification**

Image acquisition was performed as previously described (Bar-Ziv et al., 2020) . Briefly, day 1 animals were picked under a standard dissection microscope onto a solid NGM plate that contained a ~15μL drop of 100 nM sodium azide. Immobilized worms were aligned head to tail and images were captured on an Echo Revolve R4 microscope equipped with an Olympus 4x Plan Fluorite NA 0.13 objective lens, a standard Olympus FITC filter (ex 470/40; em 525/50; DM 560), and an iPad Pro for the camera and to drive the ECHO software.

To quantify fluorescence, a COPAS large particle biosorter was used as previously described (Bar-Ziv et al., 2020) . Data were collected gating for size (time of flight [TOF] and extinction) to exclude eggs and L1 animals. Data were processed by censoring events that reached the maximum peak height for Green or Extinction measurements (PH Green, PH Ext = 65532) and censoring events < 300 TOF to exclude any remaining L1 animals. All fluorescence data were normalized to TOF to account for worm size and plotted as log2(Green/TOF). Relative worm length was quantified by plotting the TOF data for each condition as a proxy for worm length.

## Reagents

**Table:** 

**Strain:**	**Genotype:**	**Available From:**
CL2166	*dvIs19[pAG15(gst-4p::GFP::NLS)] III*	CGC
